# Influence of exercise mode on pregnancy outcomes: ENHANCED by Mom project

**DOI:** 10.1186/s12884-015-0556-6

**Published:** 2015-06-09

**Authors:** Carmen Moyer, Jeffrey Livingston, Xiangming Fang, Linda E May

**Affiliations:** Department of Kinesiology, East Carolina University (ECU), Greenville, NC 27834 USA; Department of Obstetrics and Gynecology, ECU, Greenville, NC 27834 USA; Department of Biostatistics, ECU, Greenville, NC 27834 USA; Department of Foundational Sciences and Research, ECU, 1851 MacGregor Downs Rd, MS#701, Greenville, NC 27834 USA

**Keywords:** Exercise mode, Fetal heart rate, Pregnancy

## Abstract

**Background:**

The extent of the benefits of exercise training during pregnancy on maternal, fetal, and neonatal health outcomes has not been sufficiently addressed. While aerobic exercise training has been determined as safe and efficacious throughout pregnancy, the effects of other training modes on fetal health and development as well as any continued benefits for the neonate, especially with regards to cardiovascular development and function, is largely unknown. In the ENHANCED by Mom study we aim to determine the effects of different modes of exercise training (aerobic, circuit, and resistance) throughout pregnancy on childhood health by controlling individual exercise programs and assessing the effects of each on fetal and neonatal health adaptations.

**Methods/Design:**

ENHANCED by mom is a cross sectional comparison study utilizing 3 intervention groups in comparison to a control group. Participants will complete three 5 min warmup + 45 min sessions weekly from 16 weeks to 36 weeks gestation of aerobic, resistance, or circuit training, in comparison to non-exercising controls. Maternal physical measurements will occur every 4 weeks throughout the intervention period. Fetal morphometric and heart measurements will occur at 34 weeks gestation. Neonatal measurements will be acquired at birth and at 1 month, 6 months, and 12 months.

**Discussion:**

A better understanding on the effects of exercise training during pregnancy on fetal and neonatal health could have a profound impact on the prevention and development of chronic diseases such as obesity, hypertension, and diabetes.

## Background

Understanding the effects of exercise during pregnancy on the fetus and neonate is just beginning to be explored. The effects of aerobic exercise on maternal health have been long understood, with the body of research indicating the safety and efficacy of maternal exercise training regarding fetal and neonatal health is growing [1–13]. The fetus is not at risk of hypoxia or significant bradycardia during maternal exercise [4, 9, 14–16]. The neonate of exercising women is also not at risk of being born disproportionately or underweight, but is similar to women who have not exercised. Moreover, fetuses of exercising women have decreased body fat mass compared to fetuses of non-exercising mothers [6].

Previous research has shown fetal adaptations in response to maternal aerobic exercise training, including heart rate modulation and improved autonomic control [10, 13, 17]. The fetal heart adapts similar to the adult heart when exposed to exercise training. For example, fetuses of exercising women had improved cardiovascular autonomic control indicated by decreased heart rates and increased HRV, relative to those of non-exercisers [10, 13]. Further analysis demonstrated a dose–response relationship, indicating that an increase in maternal exercise intensity and time spent participating in physical activity results in a greater fetal cardiovascular adaptation (i.e. decreased fetal HR and increased fetal HRV) [13]. A recent study reported that both continuous (aerobic) and intermittent (strength) exercise training throughout pregnancy are positively correlated to fetal cardiovascular adaptations [12]. May et al. [12] observed that intermittent exercise (i.e. strength exercises) throughout pregnancy may increase fetal heart adaptability compared to continuous exercise training (i.e. aerobic exercise) throughout pregnancy [12]. While fetal HRV can be altered with exercise, the effects of maternal exercise on fetal cardiac compliance remain elusive [10, 13, 18]. Cardiac compliance improves during normal maturation of the healthy neonatal heart [19, 20]. Exercise training in childhood further contributes to these natural increases [2]. However, since the cardiovascular and endocrine response differs with exercise type (i.e. aerobic, strength), the adaptation differs accordingly. For example, aerobic exercise in children is associated with lower heart rate, blood pressure, and lower lipid levels; conversely, resistance training in children has little or no change in heart rate, blood pressure, and lipid levels [21, 23]. However, both forms are known to decrease body fat composition [22]. It is currently unknown if the fetal physiological adaptation to exercise type is similar to the response of exercise in children as a result of in utero exposure to maternal exercise.

These studies have focused primarily on maternal aerobic exercise training or self-reported physical activity [9, 10, 12, 13, 17], measuring fetal HR and HRV. A scarce amount of research exists regarding the effects of other training modes, such as resistance training or circuit training during pregnancy, on fetal health and development as well as any continued benefits for the neonate, especially with regards to cardiovascular development and function. Research to date has only determined that various modes of maternal exercise training do not present undue risk to fetal or neonatal development [2, 24–26].

The primary aim is to determine the effects of different modes of exercise training (aerobic, circuit, and resistance) throughout pregnancy on health until the first year of life. The study presented will control individual exercise programs of varying modes throughout pregnancy to assess the effects of each on fetal and neonatal health adaptations.

We hypothesize that we will find: (1) lower measurements of maternal resting heart rate, weight gain, and body fat composition in women as follows: aerobic trained group = combination training group < strength training only group < < control group; (2) lower fetal HR, and increased infant HRV, stroke volume & cardiac output at 34 weeks gestation, 1, 6, and 12 months postnatal when exposed to various modes of maternal exercise compared to fetuses of women in the control group with greatest differences in aerobic trained group = combination training group > strength training only group > control group; and (3) normalized weights with lower fetal and birth body fat measures (i.e. BMI, body fat percentage) of all exercising groups compared to controls.

The purpose of this paper is to present the design and methodology of the Enhanced Neonatal Health and Neonatal Cardiovascular Efficiency Developmentally (ENHANCED) by Mom pregnancy study.

## Methods

### Study design

**Fig. 1:**
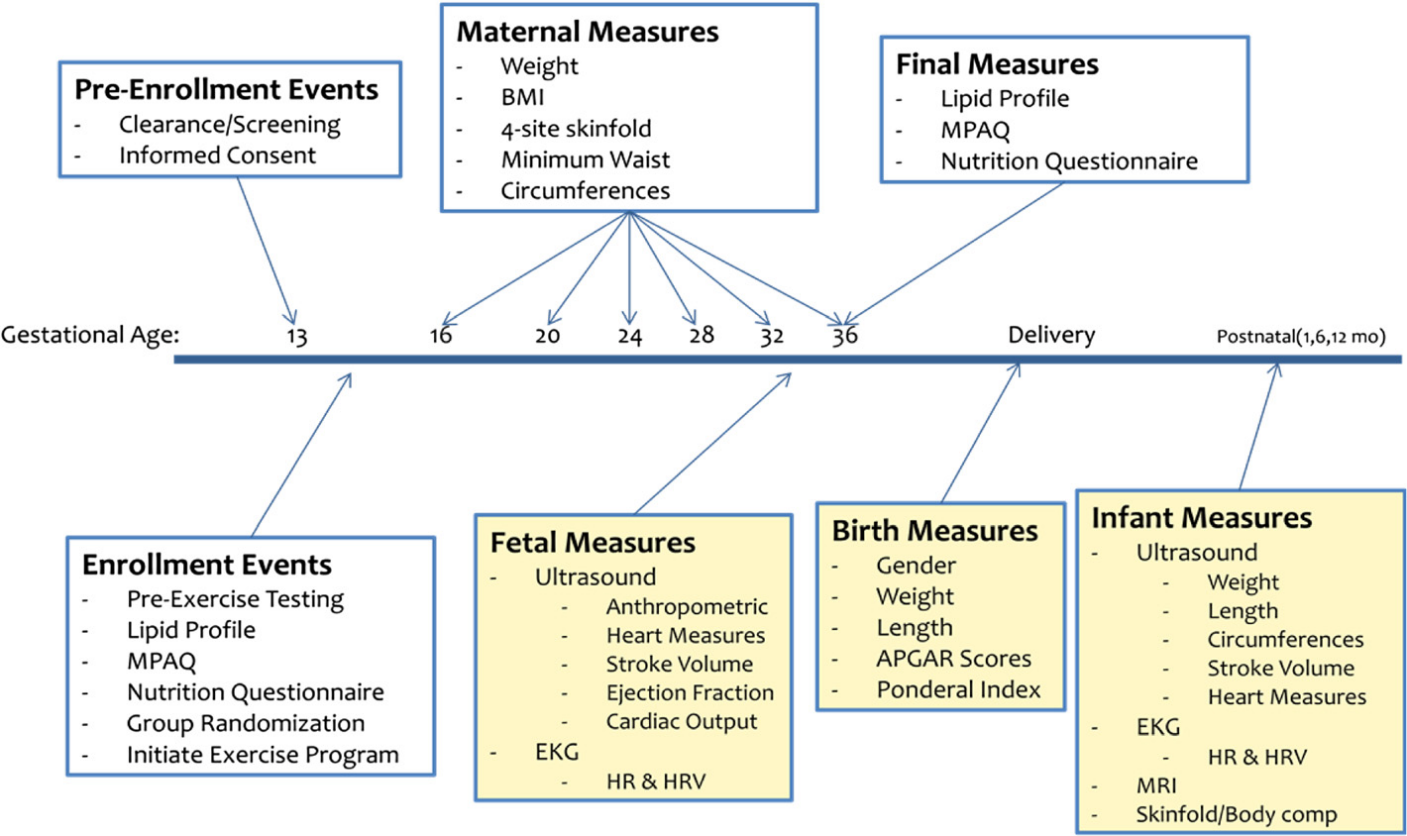
Schematic Timeline of ENHANCED by Mom project

This study will be a cross sectional comparison using convenience sampling. All protocols have been approved by the East Carolina University Institutional Review Board. Informed written consent will be obtained from each participant prior to enrollment. The intervention period will begin at 16 weeks gestation and continue to 36 weeks gestation. Participants will be screened and enrolled between 13 and 16 weeks gestation to avoid any prevalent bouts of morning sickness and spontaneous abortion, which may occur and complete a pre-intervention appointment with a supervised submaximal exercise test. A visual description of the study’s methodology is presented in Fig. 1.

### Study population

This study aims to recruit women with low risk, singleton pregnancy who have received a physician’s clearance to participate in physical activity; those that were previously sedentary or active; between the ages of 18 and 40; a pre-pregnancy body mass index (BMI) 18.5-34.9; gestational age ≤16 weeks; not currently using alcohol, tobacco, recreational drugs, or medications for mental health disorders; and not currently meeting any of the contraindications to exercise in pregnancy as outlined by the American College of Sports Medicine (ACSM) and the Society of Obstetricians and Gynecologists of Canada guidelines (Table 1) [8, 27, 30]. Participants with pre-existing diabetes, hypertension, or other cardiovascular disease are excluded from the study. Participants with comorbidities known to affect fetal growth or well-being such as systemic lupus erythematosus are excluded from participation. Any participant diagnosed with gestational diabetes mellitus (GDM) during the study will remain enrolled in the study, but their results will be analyzed separately from other participants.

**Table 1:** Contraindications to aerobic exercise during pregnancy. Adopted from *ACSM's guidelines for exercise testing and prescription [* 43]

Absolute Contraindications to Aerobic Exercise During Pregnancy	Relative Contraindications to Aerobic Exercise During Pregnancy
Hemodynamically significant heart disease	Severe anemia
Restrictive lung disease	Unevaluated maternal cardiac arrhythmia
Incompetent cervix/cerclage	Chronic bronchitis
Multiple gestation at risk for premature labor	Poorly controlled type I diabetes
Persistent second or third trimester bleeding	Extreme morbid obesity
Placenta previa after 26 weeks gestation	Extreme underweight (body mass index <12)
Premature labor during the current pregnancy	History of extremely sedentary lifestyle
Ruptured membranes	Intrauterine growth restriction in current pregnancy
Pregnancy induced hypertension	Poorly controlled hypertension/preeclampsia
	Orthopedic limitations
	Poorly controlled seizure disorder
	Poorly controlled thyroid disease
	Heavy smoker

### Study setting

Exercise testing will take place at an approved university exercise facility supervised by at least two certified (ACSM, First Aid, and CPR) staff members. In addition, exercise training sessions will be directly supervised (one-on-one) by a study staff member with an ACSM and/or personal trainer certification or who has been trained by a certified staff member on the study protocol. Exercise training will be modified within study parameters to participant preferences, choosing 3 days to attend supervised sessions at one of two approved university facilities between 7:00 a.m. and 7:00 p.m. Monday through Friday and 8:30 a.m. to 11 a.m. on Saturdays. Maternal measurements will be completed prior to exercise training at the appointed time. Local medical offices will be used for fetal ultrasounds, neonatal cardiovascular measurements, and neonatal body composition measurements.

### Recruitment

Participants will be recruited by brochures at local obstetrics clinics, as well as an email announcement to institutional faculty and staff. An inclusion and exclusion criteria card will be used by clinic staff to inform eligible patients of the study and determine individual interest in participation. These criteria will also be used by study staff to screen possible participants requesting more information regarding the study.

### Sample size

Power analysis was done based on our preliminary data and ANOVA to detect differences among the four groups post training. To ensure a power of 80 % and a significance level of 0.05 for each aim, 1) we will need a minimum of 15 participants per group (maternal HR) and a maximum of 70 per group (maternal body composition), 2) fetal and infant heart measures, we will need a minimum of 9 (fetal HR) and maximum of 21 per group (infant HRV), 3) we will need a minimum of 3 participants per group (birth weight) and a maximum of 22 per group (infant body composition). In order to detect differences minimum differences in all aims, we will need to recruit 3–70 participants per group. Therefore, we will aim for 70 per group plus an average participant attrition of 35 % inherent in long-term exercise intervention studies, we will recruit 380 participants (95 per group).

### Intervention

Upon enrollment in the study, participants will complete an initial testing appointment. Upon arrival, a non-fasting venous sample will be collected, processed, and stored for later analysis of maternal factors (i.e. blood glucose). All participants will perform submaximal VO_2_peak treadmill testing using a modified Balke protocol as validated and replication by Mottola et al. [31] in order to determine individual THR zones. In order to enhance retention, we will utilize a restricted randomization process, in which the content of each protocol will be explained to participants and they provide us with two or three group preferences of which they are then randomized into one of those groups. Participants in the circuit training and resistance training groups will complete 1 repetition maximum testing on Cybex machines after completion of the treadmill exercise test to determine appropriate external loads for exercise training. This will also be used as a time for proper instruction on the technique of each exercise used. Upon completion of pre-exercise testing, participants will complete a modified physical activity questionnaire [32] to determine pre-pregnancy activity levels and a nutrition questionnaire [33] to ensure that participants are maintaining adequate diet and nutrition levels not only for increased energy needs of pregnancy, but also for the increased energy needs of exercise. These questionnaires will be completed a second time at the end of the intervention period to verify maternal activity and nutrition levels throughout the duration of the study [29].

#### Exercise protocols

All groups, including controls, will begin each exercise session with a 5-min aerobic warm-up of low intensity (i.e. treadmill speed ≤3.0 mph) followed by their respective protocols. HR will be monitored using a Polar FS2C heart rate monitor and maintained in the THR range determined by each participant’s pre-exercise test, but not to exceed THR ranges validated for pregnant women [34]. Moderate intensity will be maintained using the Borg scale of perceived exertion [35], with the goal of 12–14 for moderate intensity, as well as the “talk test,” which means being able to converse while exercising without feeling short of breath.

The aerobic training group will perform aerobic exercise using treadmills, ellipticals, or recumbent bicycles for a 45-min workout. Treadmill speed will be maintained at ≤3.0 mph, adjusting the percent grade to maintain the individual’s pre-determined THR range. Elliptical and recumbent bike resistance and speed levels will be adjusted as necessary throughout the study’s duration to maintain the individual’s pre-determined THR zone.

The resistance training group will perform 45 min of various resistance exercises. Two to three sets of 15 repetitions will be completed at a moderate resistance per ACSM guidelines for resistance training in pregnant individuals [29], using an RPE of 12–14 to maintain moderate intensity [36]. Resistance exercises will consist of seated Cybex machine exercises including leg extension, leg curl, shoulder press, chest press, triceps extension, and latissimus dorsi pull down. Dumbbells will be used for bicep curls, lateral shoulder raises, and front shoulder raises. Resistance bands and dumbbells will also be used as an alternative method for Cybex machine exercises if the participant is unable to maintain proper form or experiences discomfort in the machine’s positioning of the body. Core exercises will be completed on an exercise ball or bench for stability or side-lying on a mat, avoid supine positioning at all times, and vary among participants based on individual levels of comfort at the present stage of pregnancy. All exercises used will be selected from safe core exercises during pregnancy based on previous research findings [37]. Participants will progressively increase resistance of each exercise according to ACSM guidelines throughout the duration of the study [29].

The circuit training group will perform a circuit of aerobic and resistance exercises, devoting equal time to each, for a 45 min workout. After the warm-up, participants will rotate between 4.5 min of aerobic and resistance training exercises, for a total of 5 circuits. Resistance exercises will be 1 set of 15 repetitions, following that of the resistance training group, while aerobic training will be similar to the aerobic training group. An RPE of 12–14 will be used to maintain moderate intensity during resistance training exercises [36]. Throughout the study’s duration, each participant will progress accordingly with changes in the resistance and speed of aerobic training exercises as well as increases in resistance of the resistance training exercises. The continual switch between resistance and aerobic training exercises will be used to maintain the participant’s HR in the appropriate THR range throughout the exercise duration to induce a training effect similar to the aerobic training only group.

The control group will have personal coaching to perform daily activities only with a maximum participation of 45 min of stretching, breathing, and flexibility exercises. Stretches will target major muscle groups of the shoulders, triceps, legs, chest, and back. Breathing exercises will combine stretches with inhalation and exhalation techniques [38, 39]. Flexibility exercises will consist of transitions between stretches with controlled breathing techniques similar to low intensity yoga training. We will monitor their HR to be maintained at a low level, below each participant’s pre-determined THR range, not to exceed exertion levels above normal activities of daily living. A combination of seated, standing, and mat exercises will be included.

### Maternal measurements

Resting HR and blood pressure will be assessed before and after each exercise session and exercise HR will be monitored throughout exercise duration to maintain individual THR zones. Maternal morphometric measurements of weight, height, body mass index, skinfolds, and circumferences will be assessed prior to the start of exercise every 4 weeks beginning at the 16^th^ week of gestation.

Serum lipid profiles will be assessed pre- and post-intervention. Blood samples will be acquired from the antecubital or cephalic vein into 1 tiger and 1 purple topped BD Vacutainer SST. After centrifugation, plasma and serum samples will be stored at −80 C° until analysis. Serum samples will be thawed overnight prior to analysis of total cholesterol and high density lipoproteins (HDL) cholesterol using a clinical blood analyzer (Beckman Coulter, version 5.3.05). All values will be measured as milligrams per deciliter (mg/dL).

Body mass index will be calculated using BodyComp software (Version 3.05). As determined by the ACSM [29], a pre-pregnancy body mass index of 18.5-24.9 will be used for the normal weight classification, 25.0-29.9 for the overweight classification, and 30.0-34.9 for the obese class I classification. Weight and height at each measurement time will be assessed in typical, casual athletic clothing without shoes. Weight will be measured in pounds to the nearest 0.1 lb on a calibrated medical scale. Height will be measured in inches to the nearest 0.25 in. Gestational weight gain will be recorded in pounds to the nearest 0.1 lb as the difference in weight during the intervention period.

Skinfold measurements will be assessed at four sites (triceps, subscapular, suprailiac, and thigh) with Harpenden skinfold calipers every 4 weeks, starting at 16 weeks gestation. All skinfolds will be assessed on the right side of the body in a standing, relaxed state according to standard ACSM procedures [29]. All measurements will be taken to the nearest 0.2 mm, with the average of 2 trials taken. If the 2 trials are not within 2.0 mm, a third will be taken to ensure accuracy of measurement. The sum of the averages of the triceps, subscapular, and suprailiac folds will then be placed into the Durnin [40] equation to estimate percent body fat as validated in pregnant populations by Miller and Ballor [41].

Circumference measurements will be assessed every 4 weeks, starting at 16 weeks gestation, using a Gulick measuring tape. All measurements will be recorded in inches to the nearest 0.25 in., with the average of 2 trials taken. If the 2 trials are not within 0.5 in., a third will be taken to ensure accuracy of measurement. The minimum waist is measured as the smallest circumference between the umbilicus and the xiphoid process. Unilateral hips/thigh, mid-thigh, calf, arm, and forearm circumferences were also measured in inches in a rotational order according to the standardized procedures established by the ACSM [29].

### Fetal measurements

At 34 weeks gestation fetal weight, body morphometrics (i.e. circumferences and bone lengths), HR and HRV, and anatomical heart measures will be assessed using fetal ultrasound during a scheduled visit at a local obstetrics clinic. All healthcare providers will be blinded to participants’ group allocation. Fetal weight is estimated in grams. Fetal morphometric measures of biparietal diameter, femur length, humerus length, head circumference, and abdominal circumference are measured in millimeters. Right and left ventricular diameters and widths are also measured in millimeters. Heart physiological measures are calculated for both the right and left ventricle based on anatomical measurements assessed in the ultrasound. Stroke volume, measured in milliliters is calculated by subtracting the end diameter in systole from the end diameter in diastole. Ejection fraction is calculated by dividing the stroke volume by the end diameter in systole and multiplying by 100 for a percentage. Cardiac output, measured in milliliters, is calculated by multiplying stroke volume and HR.

### Neonatal (Birth) measurements

Neonatal gender (M/F), gestational age (weeks), birth weight (grams), birth length (cm.), type of delivery, as well as 1 and 5 min APGAR scores will be acquired from birth records after maternal consent. Ponderal index (kg/cm^3^), a measure of neonate leanness similar to adult body mass index, is calculated by the following formula: PI = 1000 [(mass in g)/(height in cm^3^)] [42].

We will be obtained by clinic staff, who are blinded to participants’ group classification.

### Statistical analysis

Participant data not meeting adequate compliance levels of 80 % will be excluded from statistical analyses. Data from any participant diagnosed with gestational diabetes during pregnancy will be analyzed separately. Alpha level will be set *a priori* at p < 0.05 for all analyses. ANOVA will be completed first to determine initial difference among exercising and control groups for maternal, fetal, and neonatal (birth) measurements. Correlation coefficients will be used to identify potential covariates of these measures including maternal characteristics (maternal age, maternal resting HR, gestational weight gain, pre-pregnancy BMI, pre-pregnancy sedentary or active) to determine any initial difference for maternal, fetal, and neonatal measurements. lifestyle) and offspring characteristics (gender, gestational age). Analysis of covariance and repeated measures ANOVA will be used on all variables to compare among groups controlling for baseline values and potential confounders (for instance, weight status, activity status, age, etc.). Spearman correlation procedures will be used to assess relationships between maternal physical activity and fetal measures (i.e. length, circumference, HR, HRV) as well as neonatal measures. Multiple regressions will be performed for fetal and neonatal measures to determine correlations with maternal exercise measures. Repeated measures ANOVA or linear mixed models will be adopted to investigate the effect of exercise mode on infant outcomes at birth, 1, 6, and 12 months. All analyses will be completed using SPSS software (SPSS version 20, Chicago, 2009).

## Discussion

Because of the increased popularity of various modes of training among pregnant women, it is essential to identify the health benefits to the fetus and neonate, especially regarding cardiovascular function and development. This study protocol is designed to assess the effects of varying exercise modes throughout pregnancy on maternal, fetal, and neonatal health outcomes. This study aims to address many unanswered questions by determining if various modes of exercise training throughout pregnancy provide different benefits to maternal, fetal, and neonatal health in normal weight, overweight, and obese women of varying pre-pregnancy activity levels. A better understanding on the effects of exercise training on fetal cardiovascular autonomic control and function and ensuing neonatal autonomic control, cardiovascular function, and body composition could have a profound impact on in utero fetal adaptations to stress and labor as well as the prevention and development of chronic diseases such as obesity, hypertension, and diabetes.

## Abbreviations

HR: Heart rate
HRV: Heart rate variability
RPE: Rating of perceived exertion
THR: Target heart rate
ACSM: American college of sports medicine
